# Supplementary material for: Patterns of Intron Gain and Loss in Fungi
Source: PLoS Biol. 2004 Nov 30;2(12):e422. doi: 10.1371/journal.pbio.0020422 (PMC532390; doi:10.1371/journal.pbio.0020422)
Supplement: Table S1 — Also available at http://genes.mit.edu/NielsenEtAl/. (4.3 MB ZIP). [file pbio.0020422.st001.zip › NielsenEtAl/html/618.html]

AN7301.1.NCU00163.1.MG05686.1.FG02528.1


```
 CLUSTAL W (1.82) Multiple Sequence Alignments - Introns Inserted


Sequence 1: NCU00163.1	504 aa
Sequence 2: MG05686.1	505 aa
Sequence 3: FG02528.1	501 aa
Sequence 4: AN7301.1	509 aa
Alignment Length: 519 aa
Number Identitical Residues: 263 aa
Alignment Score (without introns) 13722


MG05686.1 	MTNALEAPTKHGIARLDYY0PTKFR2--~KSTDFEVHRNWLAITHSLHPWQWYYE~RTSE
NCU00163.1	MAEIYPSLVQCAIVATAFK~VLLFP~AY2KSTDFEVHRNWLAITHSLPLWEWYYE~KTSE
FG02528.1 	MSEHYPTLAQTALVAAAFK~ILLFP~AY2KSTDFEVHRNWLAITNSLPLEKWYVE~KTSE
AN7301.1  	MAELYPSLTQCAIVATAFK~ILLFP~AY2KSTDFEVHRNWLAITHSLPVQEWYYE0KTSE
          	*::   : .: .:.   :     *  :  ***************:**   :** * :***

MG05686.1 	WTLDYPPFFAYFEWVLSQVARLVDPAMLRIKNLEYDSWQTIYFQRWTVIVTELFLVYALQ
NCU00163.1	WTLDYPPFFAYFEWIMSQVARLADPAMIWVHNLEYDSWQTVYFQRWTVIVTELVLLYALQ
FG02528.1 	WTLDYPPFFAYFEWILAHVARLVDPLMVKVYNLDYDSWQTVYFQRTSVIVTELVLVWALQ
AN7301.1  	WTLDYPPFFAVFEWALSQLAQYVDPAMLDVQNLNYDSWQTVYFQRATVILSELVLFYALN
          	********** *** ::::*: .** *: : **:******:**** :**::**.*.:**:

MG05686.1 	L2VIATLRFVDSTSGSH~RRAAHAAAISILLSPALLIIDHIHFQYNGVMYGLLIASLSLA
NCU00163.1	M~YKGIL------SGSL0R-AAHAAAVSILLSPGLLIIDHIHFQYNGVMYGILIASLVLA
FG02528.1 	T~FIETA------PLKS~RRAAQTVALSIILSPGLLIIDHIHFQYNGFMYGILVMSLVLA
AN7301.1  	R2FIRSD------PQPT~KHLAHAASLSILLSPGLFIIDHIHFQYNGFLYGILVLSIVWA
          	             .    :  *::.::**:***.*:***********.:**:*: *:  *

MG05686.1 	RTKSGLLGSGFIFAALLCMKHIYAYLAPAYVVFLLRAYCLHP----KSIFRIQFFNCLKL
NCU00163.1	KKKSSLLASGLVFAALLCMKHIYLYLAPAYFVYLLRVYCLPPKLSPRSIFRIQFFNCVKL
FG02528.1 	RQNSELLSSGLIFAALLCFKHIYLYLAPAYFVYLLRAYCLSS----KSIFRIRFLNSIKL
AN7301.1  	RKQSKLLYSGIAFAVLLCLKHIYLYLAPAYFIYLLRAYCLDP----KSVFRPRFGNAFKL
          	: :* ** **: **.***:**** ******.::***.*** .    :*:** :* *..**

MG05686.1 	GGGILAIVVAAFGPFAVKGQMQQVLSRLFPFARGLCHAYWAPNVWAMYSFMDRVLIIA1A
NCU00163.1	GGGIAAIFAAAFGPFALKNQIPQIFSRLFPFSRGLCHAYWAPNVWALYSFMDRLLISL~A
FG02528.1 	GLGIGAIFGAAFGPFAAMDQIPQLLSRLFPFSRGLCHAYWAPNVWALYSFADRILIHV1A
AN7301.1  	GLSIITVFGLAFGPFVYWGQLLQLKDRLFPFSRGLCHAYWAPNVWAMYSFTDRILIPF1A
          	* .* ::.  *****.  .*: *: .*****:**************:*** **:**   *

MG05686.1 	PKLGLPVKSEALASVTRGLIGDSSFAVLPNITPRMCFVLTLVFQA~LPLARLFKKPTWDN
NCU00163.1	PRIGLPIKADALNSVTRGLVGDTSFAVLPDITPRMCFVLTLLFQA~IPLIKLFMRPTWEG
FG02528.1 	PRLGWAVNKSALQSVTRGLVGDTAFAVLPEISPRTCFVLTLFFQV~VPLIKLFFQPTWET
AN7301.1  	PKLGLPVNQDALTSVTRGLVGDTSFAILPEISKEYTFVLTLLSLI0LPLIKLWLRPDYYT
          	*::* .:: .** ******:**::**:**:*: .  *****.    :** :*: :* :  

MG05686.1 	FVGAVTLCGYASFLFGWHVHEKAILLVIIPFSLIALKDRRYLSAFRPLAVAGHVSLFPLL
NCU00163.1	FIGGVTLCGYASFLFGWHVHEKAILLVIIPFSLIALKDRRYLGAFRPLAVAGHVSLFPLI
FG02528.1 	FIGAVTLCGYSSFLFGWHVHEKAILLVIIPFSLIALRDRRHLGAFRPLVVAGYVSLFPLL
AN7301.1  	FVGALTLCGYASFLFGWHVHEKAILLIIIPFSLIALKDRRYFSAFRPIAVAGHVSLFPLL
          	*:*.:*****:***************:*********:***::.****:.***:******:

MG05686.1 	FTPAEFPIKTAYTVFWLILFLIVFDRLAPASSRPRFFLFDRLNTLYIAVSIPLIVYCSLV
NCU00163.1	FTPAEFPIKTVYTIFWLVLFLMAFDRLAPAPTRQRLFLFDRFSTAYITVSIPLIFYCSLM
FG02528.1 	FTPAEFPIKTIYTIVWLVVFLLAFGRLAPASNKPRIFLLDRFSTLYIAISIPLILYCSLL
AN7301.1  	FTAAEFPLKTVYTLLWLILFLFVFDRIAPVPERPRIFLVDRFSLLYITVSIPLILYTSLV
          	**.****:** **:.**::**:.*.*:**.. : *:**.**:.  **::*****.* **:

MG05686.1 	HQVVFDKSY~EFLPLMFTSSYTAVGVFGSWIGFMVVYFAS--------
NCU00163.1	HGIIFGKSY~EFLPLMFTSSYSAIGVVGSWLGFMVVYFTE--------
FG02528.1 	HHLIFGKAY~EFIPLMFTSSYTAIGVVGSWVGYMVVYFTA--------
AN7301.1  	HQLIFGLES2SITPSQPLEQRIPSPVTSASHSSLRFWYRMSAKASMTP
          	* ::*.    .: *    ..  .  * .:  . : .::  ::.:: :.
```
